# Observations on the Cause of the Disease Known as Sun Stroke

**Published:** 1856-06

**Authors:** Sanford B. Hunt


### ART. VI.—*Observations on the Cause of the Disease known as Sun Stroke.* By SANFORD B. HUNT, M. D.

As the season is now approaching in which we may not unreasonably expect to witness occasional cases of this sudden and terrible malady, it may be useful to recur to it now as a subject, in itself interesting, and especially so to those who have made its causation and pathology a subject of study.

The name "sun-stroke," or *coup de soliel*, implies that is produced by the direct rays of the sun, and its pathology has been almost universally conceded to be a sudden and intense congestion of the brain sufficiently severe to cause immediate death. Based on this view of the pathology, the treatment has consisted almost entirely of heroic blood-letting, and the application of cold to the head.

Unfortunately no one of these propositions or theories is proven, and perhaps it is not too much to state that no one of them is correct. It is the object of this article to consider briefly the questions involved in them, and taking them one by one, to bring to bear upon them such light as is afforded by the copious statistical tables which emanate from the health-offices of our principal cities. In conclusion, I shall endeavor to reconcile the facts of the disease so far as known, and suggest another theory of causation—one which I have already incidentally advanced on two or three occasions, but in which I have no especial claim to originality.

The first question for consideration is,

*Are the direct rays of the sun necessary to the production of the disease known as sun-stroke?*

Undoubtedly the larger number of cases occur in the open air, and in unshaded locations. But a very large number, so great as not to be considered exceptional, occur within doors or on cloudy days.

During the great *epidemic* of sun-stroke in New York, in August, 1854, of 235 deaths in that city from this cause, 49 were females. The argument here would be that as females do not live much out of doors, that some, at least, of these occurred under shelter. This supposition is confirmed by the fact that Dr. H. D. Swift, in his valuable paper on "Exhaustion from the Effects of Heat"—a monograph remarkable for the intelligence of its pathological views—mentions that "Eleven patients were attacked one morning in the laundry of one of our principal hotels; several were brought to us from a sugar refinery, where, after working several hours in a close and over-heated apartment, they fell down suddenly in a state of insensibility; and we had an opportunity of comparing their symptoms and lesions with those who became exhausted after laboring in the sun, but were unable to satisfy ourselves of any distinction."

Again, Dr. Reyburn, of St. Louis, in his "Report on the Diseases of Missouri and Iowa," made to the American Medical Association at its meeting for 1855, furnishes the following statement:

"The cases of the disease that occurred in the last summer were not all traceable to direct insolation; the furnace-tenders in engine rooms, and bakers unexposed to the sun, were sometimes attacked. One case is reported to us of a female who had not been out of the house during the entire of a very hot day, being attacked."

It is evident, then, that insolation is not essential to the creation of this disease, but that it may occur in shaded localities, and even on cloudy days, for Dr. Reyburn's statistics show that four deaths by "sun-stroke" occurred in St. Louis on the 5th of July; and on reference to Dr. Engleman's meteorological tables, I find that this day there was a very cloudy sky, with rain and thunder. Moreover this was was not a very warm day, the mean temperature being only 79°. I shall have occasion to refer to this day again, with reference to another meteorological condition.

SECONDLY.—*Is congestion of the brain the special pathological condition present in this disease?*

It is only recently that the fact has been fully recognized, that in the great majority of instances of sun-stroke, the symptoms have been those of syncope or exhaustion. Dr. Swift, in the paper before alluded to, proves from both symptoms and *post-mortem* appearances that the latter is generally the true condition. Indeed, he says distinctly, that he has seen "a few, a very few cases, of insolation verified by a *post-mortem* examination,—certainly not *one* during the past year, although examinations were made in *all the cases* in which we suspected any cerebral lesion." And he gives one case where a hot head, suffused and injected eyes, contracted pupils, swollen countenance, coma and stertorous respiration, with "fair strength" of pulse, all seemed to indicate intense cerebral congestion; but after death none was found.

So far as we know, the question of congestion of the brain rests rather upon the symptoms than the pathological evidence. In a case which we witnessed a few years since, all the symptoms of intense congestion were were present. We bled the patient freely from both arms. Soon after the flow of blood commenced he passed into the most violent convulsions, which were only discontinued on the production of profound syncope. Yet the symptoms next day did not indicate that congestion had been present. His recovery was rapid, and we have always felt some doubt as to whether congestion were really present in that apparently well-marked case.

At any rate enough is known to prove that contracted pupils and convulsions are not to be accepted as sufficient proof of congestion, and we must wait for actual *post-mortem* evidence of it to verify its existence. It is already plain that only a very limited number of cases are congestive, and it is quite probable that that limited number will be much decreased on careful study.

Dr. Swift has more correctly given the name of "Nervous Exhaustion from the Effects of Heat" to the disease. The nervous exhaustion is proven, and it is also proven that the brain is rather anæmic than congested. But the cause assigned by Dr. Swift is not satisfactory to me, and I expect to prove that heat is not alone sufficient to act as a cause.

*What are the facts in relation to heat as a cause of coup de soliel?*

During the period of greatest mortality from sun-stroke in 1853, in New York, the temperature was, according to the register kept at the New York Hospital, not very high. During August, as we have already mentioned, 235 deaths occurred from this cause. It will be interesting to examine the weather record of this period. We find from it that the temperature at 3, P. M., the hottest hour in the day, was above 90° on three days only; on which it stood respectively at 92°, 91° and 90°. There were two days only on which it stood between 85° and 90°. There were five days on which it stood between 80° and 85°, leaving twenty-one days in this month of "excessive heat," on which the temperature ranged lower than 80°. With reference to insolation we may also note that twelve days were cloudy, and that it rained on eleven of them, the total amount of rain falling being large, viz: 6.04 inches.

Turning to St. Louis we find that in the summer of 1854, during a period of nine days, (the last four of June and first five of July) fifty-three deaths occurred from sun-stroke. The mean daily temperature of this period was 86°. Subsequently in July, another period of nine days occurred, the mean temperature of which was 88°, during which only seventeen deaths occurred. If temperature is the cause why this disparity? Again four deaths occurred on the 5th of July, with a very cloudy sky, with rain and thunder, and a mean temperature of only 79°.

Here we leave the question of temperature. The facts adduced are conclusive that, though a certain temperature is necessary, neither the frequency or the fatality of the disease increase with a further rise of the thermometer.

Heat, then, is not the essential cause; neither are the direct rays of the sun necessary.

What, then, is the essential condition for the production of coup-de-soliel, or rather exhaustion from the effects of heat?

I have been led to believe, from a careful survey of the premises, that a high humidity is the essential condition. This opinion is based upon weather records and upon analogy.

In the first place the condition of nervous exhaustion is never produced by a high, yet dry heat. The various experiments going to prove that a person may live and bieathe without evil effects in a temperature high enough to cook a steak, or an egg, are too well known to need comment. Experimenters have borne for half an hour a temperature of 135°, without great discomfort.

No person, however, could live that time in a vapor-bath of that heat. The effect of surplus moisture in prostrating the nervous system, impairing the vigor of the heart's action, and producing syncope, is as well known as the vapor-bath, and needs no comment. The symptoms of coup-de-soliel, in its ordinary form, are precisely similar to prolonged syncope. The exhausting influence of a sultry day, or of a hot sun directly after a shower, are familiar instances of the effect of high humidity upon the system. But the proposition remains to be proven by hygrometrical observation, and not by the uncertain perceptions of the senses. I have purposely omitted to mention the hygrometrical condition of New York and St. Louis in the epidemics cited, because it was desired that the relation of *heat* in their causation should be tested by itself.

We may now, however, return to it.

Mr. Blodget (then of the Smithsonian Institution) said of it (New York Journal of Medicine, Oct., 1853,) that "the temperature of evaporation at New York, at the time of greatest mortality in August, was from 80° to 84°, being higher than the maximum temperature of evaporation at New Orleans at any time in 1852, by 2°. Commenting on this point on another occasion, we made the following remarks:

"This implies, necessarily, a high fraction of saturation, and placing all the evidence together—the fact that the temperature at 2, P. M., was only 90° to 92° (not an unusual heat for the season,) that the cases were mostly among foreigners, that Dr. Swift describes the symptoms as indicative of 'nervous debility,' and not of 'cerebral congestion,' that the dew-point reached a tropical maximum, and the conclusion is irresistible that, not dry heat, but a long-continued bath of aqueous vapor was the true cause of this unparalleled mortaliy. But, as if to make the evidence irrefragable, we are told that 'eleven patients were attacked one morning in the laundry of one of our principal hotels;' and 'several were brought to us from a sugar refinery, where, after working several hours in a close and over-heated apartment, they fell down suddenly in a state of insensibility.' I regard these last mentioned facts as of the last importance. Here, in a laundry, or in a sugar-refinery, unaffected by solar rays, filled with vapor artificially produced, having an excessive humidity, unventilated (for on these fatal days there was no wind,) men fell by dozens in sudden death. The experience of all time contradicts the idea that dry heat can produce these effects, and I regard them as conclusive upon the question, whether or no the combinatiou of high heat and humidity is of itself a cause of disease."

Turning again to St. Louis, we ascertain from Dr. Engleman's tables, that during the period of nine days before alluded to, the mean temperature of evaporation was 78°, against a dry bulb temperature of 86°. The fraction of saturation is high enough to prove the presence of an immense amount of aqueous vapor, nearly as much, in fact, as could be forced into the air without having it in the form of steam.

Without multiplying statistics, I may state that the examinations of such weather records, corresponding with periods of mortality from sun-stroke, as have fallen under my notice, has uniformly resulted in fixing a high temperature of evaporation as the efficient condition of the cause of the disease, and that without definite relations to the dry bulb temperature.

A question of much interest connected with this theory of causation, is as to what power a humid atmosphere exerts on the absorption of the solar rays. I made last summer, some experiments, with a view to ascertain this point. So far as they went they seemed to prove that the difference between a thermometer in the sun and one in the shade, is greatest on days of least humidity. The observations were, however, made without sufficient precaution against reflected heat, and will be repeated during the present summer with care. The method of making them is to use four thermometers: 1st, one with a dry bulb; 2d, one with a wet bulb—these two to be in the open air, shaded. A third thermometer should be exposed with a naked bulb to the sun's rays, while a fourth should be similarly exposed and wrapped around with black wool. Only clear days should be noted. In this way we hope to establish some relation between humidity and radiation, corresponding with that which is evident to the senses in the chill produced on going into the shade on a humid day.

